# Supplementary material for: Analysis of PPARγ Signaling Activity in Psoriasis
Source: Int J Mol Sci. 2021 Aug 10;22(16):8603. doi: 10.3390/ijms22168603 (PMC8395241; doi:10.3390/ijms22168603)
Supplement: Supplementary file 1 [file ijms-22-08603-s001.zip › Supplemental materials_Analysis of PPARg signaling activity in psoriasis/Pathway models/Models images and html files/Interleukin-17 and interleukin-22 signaling in psoriasis/10825.html]

SOS1


# Protein SOS1

|  |  |
| --- | --- |
| URN | urn:agi-llid:6654 |
| Total Entities | 0 |
| Connectivity | 667 |
| Name | SOS1 |
| Description | son of sevenless homolog 1 (Drosophila) |
| Notes | This gene encodes a protein that is a guanine nucleotide exchange factor for RAS proteins, membrane proteins that bind guanine nucleotides and participate in signal transduction pathways. GTP binding activates and GTP hydrolysis inactivates RAS proteins. The product of this gene may regulate RAS proteins by facilitating the exchange of GTP for GDP. Mutations in this gene are associated with gingival fibromatosis 1 and Noonan syndrome type 4. [provided by RefSeq, Jul 2008] |
| Primary Cell Localization | Cytoplasm |

---

|  |  |
| --- | --- |
| Pathway | Alzheimer's Disease |
|  | Osteoarthritis |
|  | Skin Aging |
|  | mTOR Signaling |
|  | mTOR Signaling Hyperfunction |
|  | Insulin Influence on Protein Synthesis |
|  | Insulin Influence on Glycogenesis |
|  | Thyroid Hormones Common non-Genomic Effects in Hyperthyroidism |
|  | Insulin Signaling |
|  | Thrombopoietin Receptors Signaling in Platelet Maturation |
|  | T-Cell Dependent B-Cell Activation |
|  | Natural Killer Cell Activation through C-Type Lectin-like Receptors |
|  | Natural Killer Cell Activation through ITAM-Containing Receptors |
|  | Natural Killer Cell Activation through Integrins and non-ITAM-Containing Receptors |
|  | T-Cell Maturation (Hypothesis) |
|  | Natural Killer Cell Activation |
|  | T-Cell Positive Selection and Neglect Induced Death |
|  | T-Cell Central Tolerance |
|  | CD8+ T-Cell Activation |
|  | Th1-Cell Differentiation |
|  | Treg-Cell Differentiation |
|  | Th2-Cell Differentiation |
|  | T-Cell Receptor Signaling |
|  | CXC Chemokine Receptor Signaling |
|  | CC Chemokine Receptor Signaling |
|  | Platelet Activation via Adhesion Molecules |
|  | Proplatelet Maturation |
|  | Toll-like Receptor Independent Sterile Inflammation |
|  | Basophil Activation |
|  | Eosinophil Survival by Cytokine Signaling |
|  | Mast-Cell Activation via IgE Signaling |
|  | Mast-Cells De Novo Synthesized Mediators via IgE Independent Signaling |
|  | CD16/CD14 Proinflammatory Monocyte Activation |
|  | Macrophage M2-Related Phagocytosis |
|  | anti-Inflammatory Function of Macrophage M2 Lineage |
|  | Neutrophil Recruitment and Priming |
|  | Neutrophil Degranulation via FPR1 Signaling |
|  | Neutrophil Chemotaxis |
|  | Negative Acute Phase Proteins Synthesis |
|  | Positive Acute Phase Proteins Synthesis |
|  | Vascular Smooth Muscle Cell/Pericyte Migration and Proliferation |
|  | Vascular Endothelial Cell Activation by Growth Factors |
|  | Dendritic Spine Morphogenesis and Stabilization |
|  | Nociception Expression Targets Signaling |
|  | NTSR1 Expression Targets |
|  | DRD2 Expression Targets |
|  | CHRM1 -> IL2 Production |
|  | IL6 Expression Targets -> Nociception |
|  | CHRNA7 -> NOS1 Production |
|  | Vitamine D Deficite and Dentin Formation |
|  | Th1-Cells Activation and Proliferation in Atherosclerosis |
|  | Arterial Hypertension |
|  | Endothelial Cell Dysfunction in Arterial Hypertension |
|  | Smooth Muscle Cell Dysfunction in Arterial Hypertension |
|  | Smooth Muscle Cell Dysfunction in Pulmonary Hypertension |
|  | Myocardial Remodeling |
|  | Sebocyte Proliferation in Acne Vulgaris |
|  | Inflammatory Reaction in Acne Vulgaris |
|  | Hair Follicle Keratinocyte Apoptosis |
|  | Androgens in Sebocyte Maturation |
|  | Atopic Dermatitis Onset |
|  | Mast-Cells Activation in Atopic Dermatitis |
|  | IL22 Induces Keratinocyte Proliferation in Psoriasis |
|  | Th0-Cell Aberrant Activation in Systemic Lupus Erythematosus |
|  | Th1-Cell Function in Systemic Lupus Erythematosus |
|  | Th2-Cell Function in Systemic Lupus Erythematosus |
|  | Acute Cytotoxic CD8+T-Cell Response against Melanocytes |
|  | T-Cell Cytotoxic Response against Melanocytes in Vitiligo |
|  | Humoral Immunity in Vitiligo |
|  | CD8+ T-Cell Response to Self-Determinants in Diabetes |
|  | Treg-Cell Function in Diabetes Mellitus Type 1 |
|  | Natural Killer T-Cell Roles in Diabetes Mellitus Type 1 |
|  | non-Suppressive Treg-Cell in Diabetes Mellitus Type 1 |
|  | TLR2 Signaling in Treg-Cell in Type 1 Diabetes (Animal Model) |
|  | Natural Killer Cell in Diabetes Mellitus Type 1 |
|  | Peripheral Tolerance to Autoantigens Recession in Diabetes Mellitus Type 1 |
|  | Treg-Cell Activation in Diabetes Mellitus |
|  | Donohue Syndrome Progression (Hypothesis) |
|  | Tubular Cell Dysfunction in Progressive Diabetic Nephropathy |
|  | Endothelial Cell Dysfunction in Progressive Diabetic Nephropathy |
|  | Mesangial Cell Dysfunction in Diabetic Nephropathy |
|  | Adiponectin Synthesis Declines in Insulin Resistance |
|  | Insulin Resistance in Hepatocytes |
|  | Hepatic Stellate Cells in non-Alcoholic Fatty Liver Disease |
|  | FOXO1 and SREBP-1C Role in beta-Cell Suppression (Rodent Model) |
|  | RET1 Mutation in Hyperparathyroidism |
|  | Cardiovascular Effects in Hyperthyroidism |
|  | CD4+T-Cell Response in Celiac Disease |
|  | CD8+ T-Cell Response in Celiac Disease |
|  | Celiac Disease |
|  | Cetuximab Resistance in Colorectal Cancer |
|  | Metastatic Colorectal Cancer |
|  | CagA Phosphorylation Dependent Signaling |
|  | EDN, NRG, NRTN, and GDNF/RET Signaling in Hirschsprung Disease |
|  | Th2-Cell Function in Ulcerative Colitis |
|  | T-Cell Cytotoxic Response in Ulcerative Colitis |
|  | FLT3/KIT/MLL Signaling in Leukemia |
|  | Apoptosis Block in Acute Myeloid Leukemia Cell |
|  | RARA Signaling in Acute Myeloid Leukemia (M3) |
|  | Acute Myeloid Leukemia |
|  | Myeloid Cell Differentiation Block in Leukemia |
|  | B-Cell Acute Lymphoblastic Leukemia |
|  | B-Cell Chronic Lymphocytic Leukemia |
|  | Chronic Myeloid Leukemia |
|  | Follicular Lymphoma |
|  | IL6/IGF1/VEGFA Signaling in Multiple Myeloma |
|  | Multiple Myeloma |
|  | T-Cell Acute Lymphoblastic Leukemia |
|  | Hepatocellular Carcinoma |
|  | Growth Factor Signaling in Hepatocellular Carcinoma |
|  | Pancreatic Ductal Carcinoma |
|  | Growth Factor Signaling in Pancreatic Neoplasms |
|  | Pancreatic Neuroendocrine Tumors |
|  | TGFBR Signaling in Pancreatic Neoplasms |
|  | Airway Smooth Muscle Cell High Rate Proliferation |
|  | IgE Induces Airway Smooth Muscle Cell Proliferation |
|  | Goblet-Cell Related Mucus Secretion in Asthma |
|  | Basophil Activation in Asthma |
|  | Mast-Cell Activation in Asthma |
|  | Antigen-Presenting Cell Role in Asthma |
|  | Th2-Cell Response in Asthma |
|  | Mucin Production in Goblet Airway Epithelial Cells |
|  | CFTR Facilitates Oxidative Stress in Airway Epithelium in CF |
|  | TNF and IL1B Induce Metalloproteinase Synthesis in Osteoarthritis |
|  | Steroidogenesis Impairement in Polycystic Ovary Syndrome |
|  | Polycystic Ovary Syndrome |
|  | PTPN22 Role in Rheumatoid Arthritis |
|  | Synovial Fibroblast Proliferation in Rheumatoid Arthritis |
|  | B-Cells Function in Systemic Scleroderma |
|  | Th2-Cells Function in Systemic Scleroderma |
|  | Skin Fibrosis |
|  | CD8+ T-Cell Action Impairment in HIV Type 1 Infection |
|  | CD4+ T-Cell Function Decline in HIV |
|  | CD4+ T-Cell Death in HIV |
|  | Estrogen Effects on Pregnancy Gingivitis |
|  | IL1 Receptor Antagonist (IL1RN) Deficiency (DIRA) |
|  | Cowden Syndrome |
|  | Gluten Impact on Neuronal System (Hypothesis) |
|  | Rett Syndrome |
|  | IL6 Promotes Inflammation in Muscular Dystrophies |
|  | Muscular Dystrophies |
|  | Dystrophin Glycoprotein Complex Signaling in Muscular Dystrophies |
|  | Osteoblast Function Decline in Osteoporosis |
|  | APP and Glutamate Signaling Cause Neuronal Dysfunction in Alzheimer Disease |
|  | Glioblastoma, Primary |
|  | Glioblastoma, Secondary |
|  | Astrocytoma |
|  | Glioma Invasion Signaling |
|  | Growth Factor Signaling in Neuroblastoma |
|  | Neuroblastoma |
|  | SPP1 Induced Survival of Myelin Specific T-Cells in Multiple Sclerosis |
|  | ERBB/VEGFR/Akt Signaling in Breast Cancer |
|  | IGF1R/AKT Signaling in Breast Cancer |
|  | ESR1/ERBB Positive Luminal Breast Cancer |
|  | Breast Cancer |
|  | Endometrial Cancer |
|  | Endometrioid Endometrial Cancer |
|  | Clear Cell Endometrial Cancer and Papillary Serous Endometrial Cancer |
|  | Angiogenesis in Endometriosis |
|  | Leptin Role in Endometriosis |
|  | Endometrioid Ovarian Carcinoma |
|  | Clear Cell Ovarian Carcinoma |
|  | EGFR and PTAFR Signaling in Ovarian Cancer |
|  | High-Grade Serous Ovarian Carcinoma |
|  | Low-Grade Serous Ovarian Carcinoma |
|  | Ovarian Cancer |
|  | Mucinous Ovarian Carcinoma |
|  | Subepithelial Fibroblasts in Middle Ear Cholesteatoma |
|  | Cholesteatoma |
|  | Prostate Cancer |
|  | Steroids Induced Cataract |
|  | Glutamate Mediated Excitotoxicity in Glaucoma |
|  | Cytokines Trigger Otitis Media |
|  | Mucin Hyperproduction in Goblet and Mucous Cells |
|  | Mesangial Cell Dysfunction in Glomerulonephritis |
|  | Interstitial Fibroblasts in Pyelonephritis |
|  | CD38/CD3 -> JUN/FOS/NF-kB Signaling in T-cell Proliferation |
|  | CD38/CD19 -> JUN/FOS/NF-kB Signaling in B-cell Proliferation |
|  | AREG -> CREB/CREBBP Expression Targets |
|  | AREG -> NCOR2 Expression Targets |
|  | AREG -> SMAD1 Expression Target |
|  | AREG -> HIF1A Expression Targets |
|  | AREG -> AP-1 Expression Targets |
|  | Noreadrenaline/Gq Expression Targets |
|  | Acetylcholine Expression Targets |
|  | Epinephrine/Gq Expression Targets |
|  | Dopamine/Gi Expression Targets |
|  | BTC -> AP-1/ATF/CREB Expression Targets |
|  | BTC -> EP300/ETS/ETV/SP1 Expression Targets |
|  | SELE -> ELK-SRF Signaling |
|  | CD247 Expression Targets |
|  | FCGR3A Expression Targets |
|  | CD72 -> CREB/CREBBP Expression Targets |
|  | CD8 -> ATF/CREB/CREBBP Expression Targets |
|  | CD80 -> NF-kB Expression Targets |
|  | CD86 -> NF-kB Expression Targets |
|  | CHRNA7 -> CREB Signaling |
|  | EGF -> HIF1A Expression Targets |
|  | EGF -> AP-1/ATF Expression Targets |
|  | EGF -> CREB/CREBBP/ELK/SRF/MYC Expression Targets |
|  | EGF -> TP53 Expression Targets |
|  | EGF -> NCOR2 Expression Target |
|  | EGFR -> NCOR2 Signaling |
|  | EGFR/ERBB2 -> TP53 Signaling |
|  | ERBB2/3 -> EP300/ETS/ETV/SP1 Signaling |
|  | EGFR -> SMAD1 Signaling |
|  | EGFR/ERBB2 -> HIF1A Signaling |
|  | EGFR -> AP-1/ATF2 Signaling |
|  | EGFR -> AP-1/CREB/ELK/SRF/MYC Signaling |
|  | EREG -> CREB Expression Target |
|  | EREG -> EP300/SP1 Expression Targets |
|  | EREG -> HIF1A Expression Target |
|  | EREG -> AP-1/ATF Expression Targets |
|  | Erythropoietin -> ELK/SRF Expression Targets |
|  | PECAM1 Expression Targets |
|  | NCAM1 Expression Targets |
|  | F2 -> AP-1/CREB/ELK/SRF/SP1 Expression Targets |
|  | PLG -> AP-1/CREB/ELK/SRF/SP1 Expression Targets |
|  | FGF9 -> AP-1/CREB/MYC Expression Targets |
|  | FGF10 -> AP-1/CREB/CREBBP/MYC Expression Targets |
|  | FGF1 -> AP-1/CREB/ELK/SRF/MYC Expression Targets |
|  | FGF1 -> NCOR2 Expression Target |
|  | FGF7 -> RUNX Expression Targets |
|  | FGF8 -> AP-1/CREB/MYC Expression Targets |
|  | FGF1 -> RUNX Expression Targets |
|  | FGF18 -> AP-1/CREB Expression Targets |
|  | FGF23 -> NCOR2 Expression Targets |
|  | FGF8 -> RUNX Expression Targets |
|  | FGF9 -> RUNX Expression Targets |
|  | FGF2 -> NCOR2 Expression Targets |
|  | FGF4 -> AP-1/MYC Expression Targets |
|  | FGF7 -> AP-1/CREB/CREBBP/MYC Expression Targets |
|  | FGF2 -> RUNX Expression Targets |
|  | FGF2 -> AP-1/CREB/CREBBP/ELK/SRF/MYC Expression Targets |
|  | FGFR -> RUNX2 Signaling |
|  | FGFR -> AP-1/CREB/CREBBP/ELK/SRF/MYC Signaling |
|  | Glutamate/Gq Expression Targets |
|  | GH1/GHR -> NF-kB/ELK/SRF/MYC Expression Targets |
|  | CSH1/GHR Expression Targets |
|  | HBEGF -> AP-1/ATF Expression Targets |
|  | HBEGF -> TP53 Expression Targets |
|  | HBEGF -> EP300/ETS/ETV/SP1 Expression Targets |
|  | HBEGF -> HIF1A Expression Targets |
|  | HBEGF -> CREB/MYC Expression Targets |
|  | HGF -> AP-1/CREB/ELK/SRF/MYC Expression Targets |
|  | HGFR -> AP-1/CREB/MYC Signaling |
|  | TAM Receptor Signaling Activation in Cancer |
|  | TGFB Signaling activation by Blocking of Tumor Suppressors |
|  | VEGFA Dependent Angiogenesis in Cancer |
|  | VEGF Independent Angiogenesis in Cancer |
|  | Integrins in Cancer Cell Motility, Invasion and Survival |
|  | CDH2 Activation Promotes Cancer Cell Migration and Survival |
|  | TGFB Family in Epithelial to Mesenchymal Transition in Cancer |
|  | Treg Cells Promote Immunosuppression in Cancer Immune Escape |
|  | IGF2 -> HIF1A/MYC Expression Targets |
|  | IGF1 -> ELK/SRF/HIF1A/MYC/SREBF Expression Targets |
|  | Insulin -> ELK/SRF/HIF1A/MYC/SREBF Expression Targets |
|  | NCAM1 -> CREB/ELK/SRF/MYC Signaling |
|  | PECAM -> SP1 Signaling |
|  | FcIgER -> ELK-SRF Signaling |
|  | IGF1R -> ELK/SRF/HIF1A/MYC/SREBF Signaling |
|  | InsulinR -> ELK/SRF/SREBF Signaling |
|  | FibronectinR -> AP-1/ELK/SRF/SREBF Signaling |
|  | Fibronectin Expression Targets |
|  | CTGF -> AP-1/CREB/MYC Expression Targets |
|  | CTGF -> NCOR2 Expression Target |
|  | IL6R -> CEBP/ELK/SRF Signaling |
|  | IL2R -> ELK/SRF/MYC Signaling |
|  | IL4R -> ELK/SRF/HMGY Signaling |
|  | IL16 -> AP-1 Expression Targets |
|  | IL6 Expression Targets |
|  | IL4 Expression Targets |
|  | IL2 Expression Targets |
|  | UrokinaseR -> ELK/SRF Signaling |
|  | PTPRC -> BCL6 Expression Targets |
|  | Leptin -> ELK/SRF Expression Targets |
|  | LPA Expression Targets |
|  | NRG1 -> EP300/ETS/ETV/SP1 Expression Targets |
|  | NRG1 -> CREB/CREBBP/ELK/SRF/MYC Expression Targets |
|  | NRG1 -> TP53 Expression Target |
|  | NRG1 -> AP-1/ATF Expression Targets |
|  | NRG1 -> HIF1A Expression Target |
|  | NGF -> SMAD3/NF-kB Expression Targets |
|  | NGF -> AP-1/TP53/MYC Expression Targets |
|  | NGF -> FOXO/MYCN/ELK-SRF Expression Targets |
|  | NGF -> CREB/CEBPB/MEF2A Expression Targets |
|  | PDGFB -> AP-1/CREB/MYC Expression Target |
|  | PDGFC -> CREB Expression Target |
|  | PDGFD -> AP-1 Expression Targets |
|  | PDGF -> AP-1/CREB/CREBBP/MYC Expression Targets |
|  | KIT -> MITF Signaling |
|  | PDGFR -> AP-1/MYC Signaling |
|  | AVP/Gs -> CREB/ELK/SRF/AP-1/EGR Expression Targets |
|  | NTS Expression Targets |
|  | AGT -> TP53 Expression Targets |
|  | AGT -> CREB Expression Targets |
|  | AGT -> ELK/SRF Expression Targets |
|  | AVP/Gq -> CREB/ELK/SRF/AP-1/EGR Expression Targets |
|  | PRL/GHR -> NF/kB/ELK/SRF/MYC Expression Targets |
|  | PDCD1 -> AP-1 Expression Targets |
|  | PTPRC -> BCL6 Signaling |
|  | GDNF -> HSF1 Signaling |
|  | NTRK -> AP-1/CREB/ELK/SRF/MYC/SMAD3/TP53 Signaling |
|  | GAS6 -> AP-1/CREB Expression Targets |
|  | FIGF -> AP-1 Expression Target |
|  | FIGF -> NCOR2 Expression Target |
|  | KITLG -> AP-1/CREB/CREBBP/MYC Expression Targets |
|  | GDNF -> HSF1 Expression Targets |
|  | CSF1 -> AP-1/CREB/CREBBP/MYC Expression Targets |
|  | PGF -> AP-1/CREB/CREBBP/MYC Expression Targets |
|  | FLT3LG -> AP-1/CREB/CREBBP Expression Targets |
|  | Ras-GRF Regulation Signaling |
|  | Androgen Receptor non-Genomic Signaling |
|  | FOXO1 Signaling |
|  | MERTK Signaling |
|  | TGF-beta Signaling |
|  | AXL Receptor Tyrosine Kinase Signaling |
|  | KRAS Signaling |
|  | Estrogens/ESR1 non-Genomic Signaling |
|  | EGFR Signaling |
|  | HRAS Signaling |
|  | TCR -> AP-1 Expression Targets |
|  | T-Cell Receptor -> AP-1 Signaling |
|  | TGFA -> TP53 Expression Targets |
|  | TGFA -> HIF1A Expression Targets |
|  | TGFA/AP-1/ATF Expression Targets |
|  | TGFA -> CREB/CREBBP/ELK-SRF/MYC Expression Targets |
|  | LTA Expression Targets |
|  | TNF -> AP-1 Expression Targets |
|  | TNF -> ELK-SRF Expression Targets |
|  | TNF -> CREB Expression Targets |
|  | TNF -> TP53/ATF Expression Targets |
|  | TNFRSF1A -> AP-1/ATF/TP53 Signaling |
|  | TNFRSF1A -> CREB/ELK-SRF Signaling |
|  | Thrombopoietin -> SPI1 Expression Targets |
|  | Thrombopoietin -> AP-1/CREB/CREBBP/MYC Expression Targets |
|  | Thrombopoietin -> SP1 Expression Targets |
|  | ROS in Angiotensin Mediated Cardiovascular Remodeling and Hypertrophy |
|  | PLAU -> ELK-SRF/AP-1 Expression Targets |
|  | VEGFA -> ATF/CREB/ELK-SRF Expression Targets |
|  | VEGFA -> NFATC Expression Targets |
|  | VEGFC -> ATF Expression Target |
|  | VEGFA -> AP-1/CREBBP/MYC Expression Targets |
|  | VEGFA -> NCOR2 Expression Target |
|  | VEGFR -> NFATC Signaling |
|  | VEGFR -> AP-1/CREB/MYC Signaling |
|  | VEGFR -> ATF/CREB/ELK-SRF Signaling |
|  | New Pathway |
|  | 1\_Inflammation in keratinocytes in the pathology of acne |
|  | 2\_Hyperseborrhea in acne vulgaris |
|  | 2\_Acute phase initiation in atopic dermatitis |
|  | 2\_Interleukin-17 and interleukin-22 signaling in psoriasis |
|  | 1\_The onset of atopic dermatitis |
|  | 1\_TNF and IL1B provoke ECM degradation in osteoarthritis |
|  | 2\_Osteoblast-mediated bone demineralization |
|  | 3\_The role of interstitial fibroblasts in pyelonephritis |
|  | 1\_Local estrogen production stimulates ectopic endometrium proliferation |
|  | 3\_Enhanced angiogenesis in endometriosis |
|  | 2\_The intense proliferation and contractile activity of smooth muscle cells in pulmonary hypertension |
|  | 1\_Th2 cell response in asthma |
|  | 3\_1\_Mucus accumulation in asthma |
|  | 2\_2\_Eosinophilia and neutrophilia in asthma: eosinophil apoptosis |
|  | 4\_Mucin hyperproduction in goblet and mucous cells in COPD |
|  | 1\_CD4+ T cell and epithelial cell response in celiac disease |
|  | 2\_CD8+ T-cell response and mucosal damage in celiac disease |
|  | 1\_2\_Insulin synthesis and secretion: insulin secretion |
|  | 1\_1\_Insulin synthesis and secretion: insulin processing and storage |
|  | 4\_1\_Insulin resistance: insulin signaling |
|  | 3\_Islet beta-cell destruction in T1D |
|  | 2\_1\_General pathways of thyroid hormone signaling |
|  | Interleukin-17 and interleukin-22 signaling in psoriasis |

---

|  |  |
| --- | --- |
| Group | Proteins Involved in Cowden Syndrome |
|  | Genes Associated with Syndromic Epilepsy |

---

|  |  |
| --- | --- |
| MedScan ID | 6654 |

---

|  |  |
| --- | --- |
| LocusLink ID | 6654 |
|  | 20662 |
|  | 313845 |

---

|  |  |
| --- | --- |
| Alias | mSos1 |
|  | son of sevenless homolog 1 |
|  | alternate sos1 |
|  | SOS1 |
|  | hSos1 |
|  | ras guanine exchange factor SOS |
|  | OTTHUMP00000128306 |
|  | rSos |
|  | Son of sevenless 1 |
|  | RIKEN cDNA 4430401P03 gene |
|  | hereditary gingival fibromatosis-1 |
|  | RasGEF SOS |
|  | Sos1 Ras guanine exchange factor |
|  | son of sevenless (Drosophila) homolog 1 |
|  | rSos1 |
|  | OTTHUMP00000204121 |
|  | gingival fibromatosis, hereditary, 1 |
|  | hereditary gingival fibromatosis 1 |
|  | SOS-1 |
|  | SOS gene |
|  | hSos |
|  | son of sevenless homolog 1 (Drosophila) |
|  | gingival fibromatosis gene |
|  | 4430401P03Rik |
|  | 4430401P03 |
|  | 9630010N06 |
|  | gingival fibromatosis protein |
|  | Son of sevenless I |
|  | GGF I |
|  | Son of sevenless |
|  | AI449023 |
|  | Sos |
|  | SOS Ras/Rac guanine nucleotide exchange factor 1 |
|  | GGF1 |
|  | hereditary gingival fibromatosis-I |
|  | Sos I |
|  | hereditary gingival fibromatosis I |
|  | son of sevenless homolog |
|  | son of sevenless proteins |
|  | son of sevenless (Drosophila) homolog I |
|  | son of sevenless homolog I |
|  | mSos |
|  | GINGF |
|  | GF1 |
|  | HGF |
|  | NS4 |
|  | guanine nucleotide exchange factor |
|  | guanine nucleotide releasing factor |
|  | ras guanine exchange factor |
|  | son-of-sevenless 1 |
|  | SOS protein |

---

|  |  |
| --- | --- |
| GO ID | 0003677 |
|  | 0005096 |
|  | 0005088 |
|  | 0005089 |
|  | 0017124 |
|  | 0005085 |
|  | 0046982 |
|  | 0001782 |
|  | 0038128 |
|  | 0038095 |
|  | 0007186 |
|  | 0000165 |
|  | 0007265 |
|  | 0007411 |
|  | 0048514 |
|  | 0003209 |
|  | 0019221 |
|  | 0007173 |
|  | 0061029 |
|  | 0001942 |
|  | 0061384 |
|  | 0008286 |
|  | 0050900 |
|  | 1904693 |
|  | 0035264 |
|  | 0048011 |
|  | 0003344 |
|  | 0043547 |
|  | 0043065 |
|  | 0045742 |
|  | 0051057 |
|  | 0035023 |
|  | 0033081 |
|  | 0042129 |
|  | 2000973 |
|  | 0051056 |
|  | 0060021 |
|  | 0007165 |
|  | 0007296 |
|  | 0005737 |
|  | 0005829 |
|  | 0043025 |
|  | 0000786 |
|  | 0005886 |
|  | 0014069 |
|  | 0008543 |
|  | 0003007 |
|  | 0002260 |
|  | 0046579 |
|  | 0007264 |
|  | 0043234 |
|  | 0005100 |
|  | 0097190 |
|  | 0007596 |
|  | 0045087 |
|  | 0030168 |
|  | 0032321 |
|  | 0032320 |
|  | 0005622 |
|  | 0032318 |
|  | 0005543 |
|  | 0006915 |
|  | 0050790 |
|  | 0008624 |

---

|  |  |
| --- | --- |
| KEGG ID | hsa:6654 |
|  | mmu:20662 |
|  | rno:313845 |

---

|  |  |
| --- | --- |
| Organism | Homo sapiens {Organism urn:agi-taxid:9606} |
|  | Mus musculus {Organism urn:agi-taxid:10090} |
|  | Rattus norvegicus {Organism urn:agi-taxid:10116} |
|  | Homo sapiens |
|  | Mus musculus |
|  | Rattus norvegicus |

---

|  |  |
| --- | --- |
| Mouse chromosome position | 17 50.67 cM |
|  | 17 44.0 cM |

---

|  |  |
| --- | --- |
| OMIM ID | 182530 |
|  | 135300 |
|  | 610733 |
|  | 163955 |

---

|  |  |
| --- | --- |
| Rat chromosome position | 6q11 |

---

|  |  |
| --- | --- |
| Hugo ID | 11187 |
|  | HGNC:11187 |

---

|  |  |
| --- | --- |
| Human chromosome position | 2p22.1 |
|  | 2p21 |
|  | 2p22-p21 |

---

|  |  |
| --- | --- |
| Swiss-Prot Accession | Q07889 |
|  | G5E9C8 |
|  | Q07889.1 |
|  | Q3USK4 |
|  | Q62245 |
|  | Q62245.2 |
|  | D4A3T0 |
|  | A8K2G3 |
|  | B4DXG2 |
|  | Q62244 |
|  | Q53SF8 |
|  | Q59GG9 |
|  | Q8NIA3 |
|  | Q9UKX9 |
|  | Q2M4G6 |
|  | Q9JKM6 |
|  | Q9QYS7 |
|  | Q497A5 |

---

|  |  |
| --- | --- |
| PIR ID | A37488 |
|  | S25715 |
|  | S25716 |

---

|  |  |
| --- | --- |
| GenBank ID | NC\_000002 |
|  | NM\_005633 |
|  | NP\_005624 |
|  | XM\_005264515 |
|  | XP\_005264572 |
|  | XM\_011533062 |
|  | XP\_011531364 |
|  | XM\_011533064 |
|  | XP\_011531366 |
|  | NG\_007530 |
|  | AC019171 |
|  | AAX93186 |
|  | AC092672 |
|  | AAY24202 |
|  | AF106953 |
|  | AAF04460 |
|  | AH011624 |
|  | AAM22406 |
|  | CH471053 |
|  | EAX00350 |
|  | EAX00351 |
|  | EAX00352 |
|  | EAX00353 |
|  | HI376402 |
|  | CBX55730 |
|  | LS482418 |
|  | SPT35795 |
|  | AB209140 |
|  | BAD92377 |
|  | AK290228 |
|  | BAF82917 |
|  | AK301960 |
|  | BAG63374 |
|  | AL833457 |
|  | AV702692 |
|  | L13857 |
|  | AAA35913 |
|  | Q07889 |
|  | NC\_000083 |
|  | NM\_009231 |
|  | NP\_033257 |
|  | AC161447 |
|  | AF233591 |
|  | AAF42937 |
|  | AH008383 |
|  | AAF08009 |
|  | AY902349 |
|  | AAY21059 |
|  | CH466537 |
|  | EDL38526 |
|  | AK014468 |
|  | AK047849 |
|  | AK140310 |
|  | BAE24327 |
|  | AK141710 |
|  | AK163885 |
|  | CB521016 |
|  | Z11578 |
|  | CAA77665 |
|  | Q62245 |
|  | NC\_005105 |
|  | NM\_001100716 |
|  | NP\_001094186 |
|  | AC\_000074 |
|  | AABR07062682 |
|  | AABR07062683 |
|  | AABR07062684 |
|  | AAHX01041857 |
|  | AAHX01041858 |
|  | AAHX01041859 |
|  | AAHX01041860 |
|  | AAHX01041861 |
|  | AAHX01041862 |
|  | CH473947 |
|  | EDM02758 |
|  | BC100642 |
|  | AAI00643 |
|  | XM\_011533063 |
|  | XP\_011531365 |
|  | XM\_011533061 |
|  | XP\_011531363 |
|  | XM\_011533065 |
|  | XP\_011531367 |
|  | XM\_011533060 |
|  | XP\_011531362 |
|  | XM\_011533066 |
|  | XP\_011531368 |
|  | NC\_018913 |
|  | AF441466 |
|  | AF441467 |
|  | AF441468 |
|  | AF441469 |
|  | AF441470 |
|  | AF441471 |
|  | AF441472 |
|  | AF441473 |
|  | AF441474 |
|  | AF441475 |
|  | AF441476 |
|  | AF441477 |
|  | AF441478 |
|  | AF441479 |
|  | AF441480 |
|  | AF441481 |
|  | AF441482 |
|  | AF441483 |
|  | AF441484 |
|  | AF441485 |
|  | AMYH02003635 |
|  | AMYH02003636 |
|  | AMYH02003637 |
|  | BC140215 |
|  | AAI40216 |
|  | AC\_000039 |
|  | AAHY01135073 |
|  | AAHY01135074 |
|  | BC146288 |
|  | AAI46289 |
|  | BC148799 |
|  | AAI48800 |
|  | AC\_000134 |
|  | ABBA01075022 |
|  | ABBA01075023 |
|  | ABBA01075024 |
|  | ABBA01075025 |
|  | AABR06041446 |
|  | AABR06041447 |
|  | AABR06041448 |
|  | AABR06041449 |
|  | AABR06041450 |
|  | AABR06041451 |
|  | AABR06041452 |
|  | NT\_022184 |
|  | NW\_927719 |
|  | NW\_001838769 |
|  | AC\_000045 |
|  | Q53SF8 |
|  | Q59GG9 |
|  | Q8NIA3 |
|  | Q9UKX9 |
|  | NT\_039649 |
|  | NW\_001030622 |
|  | AF094674 |
|  | AF094675 |
|  | AF094676 |
|  | AF094677 |
|  | AF094679 |
|  | Q2M4G6 |
|  | Q3USK4 |
|  | Q9JKM6 |
|  | Q9QYS7 |
|  | NW\_047754 |
|  | NW\_001084845 |
|  | Q497A5 |

---

|  |  |
| --- | --- |
| Swiss-Prot ID | SOS1\_HUMAN |
|  | SOS1\_MOUSE |
|  | Q3USK4\_MOUSE |
|  | A8K2G3\_HUMAN |

---

|  |  |
| --- | --- |
| Cell Localization | Cytoplasm |

---

|  |  |
| --- | --- |
| Ensembl ID | ENSG00000115904 |
|  | ENSP00000384675.2 |
|  | ENST00000402219.6 |
|  | ENSP00000378479.2 |
|  | ENST00000395038.6 |
|  | ENSMUSG00000024241 |
|  | ENSMUSP00000067786.5 |
|  | ENSMUST00000068714.6 |
|  | ENSRNOG00000007106 |
|  | ENSRNOP00000009359.6 |
|  | ENSRNOT00000009359.7 |
|  | ENSP00000384675 |
|  | ENST00000402219 |
|  | ENSP00000378479 |
|  | ENST00000395038 |
|  | ENSMUSP00000067786 |
|  | ENSMUST00000068714 |
|  | ENSRNOP00000009359 |
|  | ENSRNOT00000009359 |

---

|  |  |
| --- | --- |
| MGI ID | MGI:98354 |
|  | 98354 |

---

|  |  |
| --- | --- |
| RGD ID | 1310949 |

---

|  |  |
| --- | --- |
| Unigene ID | Hs.709893 |
|  | Hs.732497 |
|  | Mm.60975 |
|  | Mm.360004 |
|  | Rn.91844 |
|  | Mm.434583 |

---

|  |  |
| --- | --- |
| Homologene ID | 4117 |

---

|  |  |
| --- | --- |
| IPI ID | IPI00020131 |
|  | IPI00311611 |
|  | IPI00873613 |
|  | IPI00767217 |
|  | IPI00360064 |

---
